# A Proteomic View of *Salmonella* Typhimurium in Response to Phosphate Limitation

**DOI:** 10.3390/proteomes6020019

**Published:** 2018-04-25

**Authors:** Jiezhang Jiang, Kaiwen Yu, Linlu Qi, Yanhua Liu, Sen Cheng, Mei Wu, Zhen Wang, Jiaqi Fu, Xiaoyun Liu

**Affiliations:** Institute of Analytical Chemistry and Synthetic and Functional Biomolecules Center, College of Chemistry and Molecular Engineering, Peking University, 202 Chengfu Rd, Haidian District, Beijing 100871, China; jiangjiezhang@pku.edu.cn (J.J.); kwyu@pku.edu.cn (K.Y.); qilinlu1987@126.com (L.Q.); liuyanhua@pku.edu.cn (Y.L.); chengsen@pku.edu.cn (S.C.); wumei@pku.edu.cn (M.W.); zhenw@pku.edu.cn (Z.W.); fujiaqi@pku.edu.cn (J.F.)

**Keywords:** *Salmonella* Typhimurium, phosphate starvation, the PhoR-PhoB two-component system, the *nag* operon

## Abstract

*Salmonella enterica* serovar Typhimurium (*S.* Typhimurium), an important foodborne pathogen, often encounters phosphate (P_i_) shortage both in the environment and inside host cells. To gain a global view on its physiological responses to P_i_ starvation, we performed proteomic profiling of *S.* Typhimurium upon the shift from P_i_-rich to P_i_-low conditions. In addition to the Pho regulon, many metabolic processes were up-regulated, such as glycolysis, pentose phosphate pathway, pyrimidine degradation, glycogen, and trehalose metabolism, allowing us to chart an overview of *S.* Typhimurium carbon metabolism under P_i_ starvation. Furthermore, proteomic analysis of a mutant lacking *phoB* (that encodes a key regulator of P_i_ shortage response) suggested that only a small subset of the altered proteins upon P_i_ limitation was PhoB-dependent. Importantly, we present evidence that *S.* Typhimurium *N*-acetylglucosamine catabolism was induced under P_i_-limiting conditions in a PhoB-dependent manner. Immunoblotting and β-galactosidase assays demonstrated that PhoB was required for the full activation of NagB, a key enzyme of this pathway, in response to low P_i_. Thus, our study reveals that *N*-acetylglucosamine catabolism may represent an additional PhoB-regulated pathway to tackle bacterial P_i_ shortage.

## 1. Introduction

As an essential element for life, phosphorus participates in many important cellular processes such as DNA and RNA synthesis, energy metabolism, membrane integrity and signal transduction [1]. Therefore, it is critical to maintain cellular phosphorus homeostasis. In bacteria, the most assimilable form of phosphorus is inorganic phosphate (P_i_) [2,3]. Many bacteria have evolved elaborate mechanisms to sense and respond to the level of phosphate in the surrounding medium. A prominent mechanism of P_i_ management is the Phosphate (Pho) regulon, which is controlled by a two-component regulatory system (TCS) [3]. While nomenclatures differ among diverse bacteria, in *Escherichia coli* (*E. coli*) this regulatory circuit is called the PhoR-PhoB TCS. TCS is often composed of a histidine kinase (e.g., PhoR) and a response regulator (e.g., PhoB) [4]. Under P_i_-limiting conditions, PhoR is activated and phosphorylates PhoB, which in turn drives the expression of many downstream genes by binding to specific DNA sequences (known as the Pho box). 

*Salmonella enterica* serovar Typhimurium (*S.* Typhimurium) is a foodborne intracellular bacterial pathogen that causes acute gastroenteritis [5]. Upon infection of host cells, *S.* Typhimurium can survive and replicate within a membrane-bound compartment called *Salmonella*-containing vacuoles (SCVs) [6,7]. To support intracellular growth, it is often indispensable for *S.* Typhimurium to tailor its metabolic pathways within the host niche. By using high-throughput proteomic approaches, we previously characterized protein expression of intracellular *S.* Typhimurium at distinct stages of infection, revealing extensive bacterial adaptations to host epithelial cells [8,9,10]. A salient feature of such adaptations is the induction of P_i_ uptake and utilization pathways, consistent with P_i_ deficiency within SCVs.

Thus far, most of our knowledge of bacterial responses to P_i_ starvation has been gathered from the studies of *E. coli* [11,12,13,14]. In particular, the Pho regulon has been extensively characterized due to its central role in managing P_i_ homeostasis. It contains at least 40 members, including the high-affinity P_i_ uptake system PstSCAB-PhoU [15]. Upon binding to PstS in the periplasm, P_i_ can be transported into bacterial cytoplasm through a channel formed by PstA and PstC while the ATP-dependent permease PstB provides the energy required for transport [3,4,16,17,18,19]. PhoU is required for dephosphorylation of PhoB in an unknown manner when P_i_ becomes sufficient. Though expected to share some features with its *E. coli* counterpart, *S.* Typhimurium physiological responses to P_i_ starvation are less understood in particular on a systems level. 

Herein we reported a proteomic survey of *S.* Typhimurium upon the shift from P_i_-sufficient to P_i_-limiting conditions. Among approximately 1600 detected proteins, we found differential regulation of 389 bacterial proteins. To differentiate PhoB-regulated and PhoB-independent pathways, we further analyzed the proteome of a *phoB*-deleting mutant in comparison to that of its parental strain. Interestingly, most of the altered proteins under P_i_ starvation are independent of the PhoR-PhoB regulatory system. Importantly, two enzymes involved in *N*-acetylglucosamine catabolism, NagA and NagB, were induced upon P_i_ starvation in a PhoB-dependent manner. Immunoblotting and β-galactosidase assays were exploited to further establish the regulation of Nag by PhoB. In summary, our proteomic work provides a global view of *S.* Typhimurium adaptation mechanisms during P_i_ starvation and identifies Nag as additional members of the PhoB regulon, which could aid in the future development of new treatment strategies for this bacterial pathogen.

## 2. Materials and Methods

### 2.1. Bacterial Strains and Culture Conditions

The *Salmonella enterica* serovar Typhimurium wild-type (WT) strain 14028s was kindly provided by Feng Shao’s laboratory (National Institute of Biological Sciences, Beijing, China). All of *S.* Typhimurium deletion mutants and strains chromosomally expressing 3 × FLAG-tagged proteins were generated by the λ-red recombination system as described previously [20,21]. A kanamycin-resistant fragment was introduced in those strains either at the site of deleted genes or after the FLAG tag. For constructing the complementation strain that harbors a copy of plasmid-borne *phoB* in the Δ*phoB* background (Δ*phoB* + pPhoB), the *phoB* fragment was inserted into a plasmid with an arabinose-inducible promoter. For constructing the strains used in β-galactosidase assays, the upstream regions of *nagB* or *pstS* were cloned and inserted into the upstream of *lacZ* in a single copy plasmid pNN387 [22]. All bacterial strains were maintained at −80 °C in Luria-Bertani (LB) broth supplemented with 25% (*v*/*v*) glycerol. A single colony was inoculated into 3 mL of MOPS (morpholinepropanesulfonic acid) medium with 1 mM phosphate (P_i_^+^) [23] and grown overnight at 37 °C with shaking. The overnight culture was then subcultured (diluted 1:20 into 3 mL of MOPS medium) with 1 mM phosphate and harvested when the optical density at 600 nm (OD_600_) reached approximately 0.5. For phosphate starvation, bacteria were first subcultured in P_i_^+^ MOPS medium till OD_600_ reached 0.3. Then cells were washed twice and resuspended in MOPS medium without phosphate (P_i_^−^). The culture was harvested when OD_600_ reached 0.5. 

### 2.2. SDS-PAGE, in-Gel Protein Digestion and Stable Isotope Dimethyl Labeling

Bacterial pellets were resuspended in the SDS sample buffer and then boiled for 5 min. Approximately 100 μg of proteins were loaded onto 10% SDS-PAGE and separated into eight fractions (see Supplementary Figure S1). In-gel protein digestion was performed as previously reported [24]. Briefly, gel slices were cut into approximately 1 mm^3^ cubes and destained with 50% acetonitrile (ACN) in 50 mM triethyl ammonium bicarbonate (TEAB), then dehydrated with pure ACN. After vacuum dehydration, protein digestion was performed in a buffer containing 1.2 ng/μL trypsin in 50 mM TEAB (10% ACN) at 37 °C overnight. Tryptic peptides were extracted from gel cubes twice by incubating with 50% ACN and 5% formic acid (FA) for 20 min at 37 °C. Finally, extracted peptides were pooled and vacuum dried for stable isotopic labeling.

The dimethyl labeling experiments were performed as previously described [25]. Tryptic peptides were resuspended in 100 μL of the reaction buffer (100 mM TEAB) followed by the addition of 4 μL of 0.6 M sodium cyanoborohydride (NaBH_3_CN). Peptides from the WT strain cultured in P_i_-limiting conditions were labeled with 4 μL 4% formaldehyde (CH_2_O) while peptides from the WT strain cultured in P_i_-rich conditions and the Δ*phoB* mutant were labeled with the same amount of deuterated formaldehyde (CD_2_O), respectively. The reaction mixture was vortexed and incubated for 1 h at room temperature. The reaction was stopped by sequential addition of 16 μL of 1% (*v*/*v*) ammonia and 8 μL of formic acid. Finally, light- and heavy-labeled peptides were pooled and vacuum dried for further mass spectrometric analyses.

### 2.3. Nanoflow LC-MS/MS Analyses

LC-MS/MS experiments were performed on a nano-LC (EASY-nLC 1200, Thermo Scientific, Waltham, MA, USA) coupled with a hybrid ion trap-Orbitrap mass spectrometer (Orbitrap Velos, Thermo Scientific, USA). The capillary column (75 μm × 150 mm) with a laser-pulled electrospray tip (Model P-2000, Sutter Instruments, Novato, CA, USA) was home-packed with 4 μm, 100 Å Magic C18AQ silica-based particles (Michrom BioResources Inc., Auburn, CA, USA). Peptide samples were reconstituted in Buffer A (described below) and approximately 200 ng of samples were loaded onto the analytical column in a single LC-MS/MS run. The mobile phase comprised of Buffer A (97% H_2_O, 3% ACN, and 0.1% FA) and Buffer B (100% ACN and 0.1% FA). The LC separation was carried out with the following gradient: Buffer B was started at 7% for 3 min, and then raised to 35% over 120 min. Subsequently, Buffer B was rapidly increased to 90% in 2 min and maintained for 10 min before 100% Buffer A was used for column equilibration. The mass spectrometer was operated in a data-dependent mode. One full MS scan (*m*/*z* 350–1500) was acquired by the Orbitrap mass analyzer with *R* = 60,000 and followed by fragmentation of the ten most intense ions in the ion trap under collision-induced dissociation (CID). Dynamic exclusion was enabled with repeat duration of 30 s and exclusion duration of 12 s.

### 2.4. Proteomic Data Processing and Bioinformatics Analysis

Raw data were searched against the *S*. Typhimurium 14028s protein database (5472 sequences, downloaded from UniProt) using the Andromeda search engine included in MaxQuant [26]. The precursor mass tolerance was set at 20 ppm and the fragment mass tolerance was set at 0.8 Da. Trypsin was selected as the digestive enzyme with a maximum of two missed cleavages. Dimethyl (K, N-term) and dimethyl (D_4_K, D_4_N-term) were set as variable modifications for light (L)- and heavy (H)-labeled samples, respectively. Oxidation (M) was set as a variable modification as well. Both peptide and protein assignments were filtered to achieve a false discovery rate (FDR) <1%. Peptides used for protein quantification were set to razor and unique peptides. Only peptides that were identified in all three biological replicates were used for protein quantification. Normalized heavy to light (H/L) ratios were calculated by MaxQuant and further processed by the Perseus software (version 1.5.4.1). Potential contaminants and the hits from the reverse database search were excluded. The *p*-values were obtained by using the one-sample two-tailed Student’s *t*-test. Proteins with abundance ratios (H/L) >2.0 or <0.5 and *p*-values < 0.05 were considered as significant differences between samples.

For the analysis of protein-protein interactions, differentially expressed proteins were searched against the STRING database (Search Tool for the Retrieval of Interacting Genes/Protein, http://string-db.org/) with the highest confidence score (score > 0.9). Only the interaction network with at least three proteins was shown, and the unconnected proteins or clusters with two proteins were not presented.

### 2.5. Bacterial Growth Competition Assays

Overnight cultures of WT and kanamycin-resistant Δ*otsB* strains were washed twice and resuspended in P_i_^−^ MOPS medium with a final OD_600_ of 0.3. Then equally mixed cultures were further inoculated for 3 hours before plating on LB agar plates with and without the supplementation of 20 μg/mL kanamycin, respectively. The plates were incubated at 37 °C for 24 h. The number of viable bacteria in the original cultures was determined by colony-forming unit (CFU) assays. The competitive index is defined as the number of viable WT bacteria divided by that of mutant bacteria. Results are presented as the mean of three independent experiments.

### 2.6. Western Blot Analysis

A *Salmonella* strain expressing 3 × FLAG-tagged NagB was cultured under either the P_i_^+^ or P_i_^−^ media as described above. Gel-separated bacterial proteins were transferred to the PVDF membrane and probed with primary antibodies specific for FLAG (Sigma, St. Louis, MO, USA) (1:5000) and anti-mouse HRP-conjugated secondary antibodies (Sigma, St. Louis, MO, USA) (1:5000). As a loading control, DnaK was probed by using *Salmonella* anti-DnaK (Enzo Life Sciences, New York, NY, USA) (1:5000) and anti-mouse HRP-conjugated secondary antibodies (Sigma, St. Louis, MO, USA) (1:5000).

### 2.7. β-Galactosidase Activity Assays

β-galactosidase activity assays were performed as previously described [10]. Briefly, *Salmonella* strains were cultured in the P_i_^−^ media as described above. Bacterial pellets from 3 mL of cultures were resuspended in 1.2 mL of Z buffer (0.06 M Na_2_HPO_4_, 0.04 M NaH_2_PO_4_, 0.01 M KCl, and 0.001 M MgSO_4_) with freshly added 50 mM β-mercaptoethanol. 30 μL of chloroform and 15 μL of 0.1% SDS were added and mixed by vortexing. The assays were started by the addition of 240 μL of 4 mg/mL *O*-nitrophenyl-d-galactopyranoside (ONPG). Upon the observation of a faint yellow color, the reaction was quenched by the addition of 600 μL of 1 M Na_2_CO_3_ and the reaction time was noted. Finally, samples were centrifuged at 14,000× *g* for 2 min, and the OD_420_ of the culture supernatant was recorded. Assay units were calculated as 1000 × OD_420_/(OD_600_) (total time). 

## 3. Results

### 3.1. Proteomic Analysis of S. Typhimurium in Response to P_i_ Starvation

**Figure 1:**
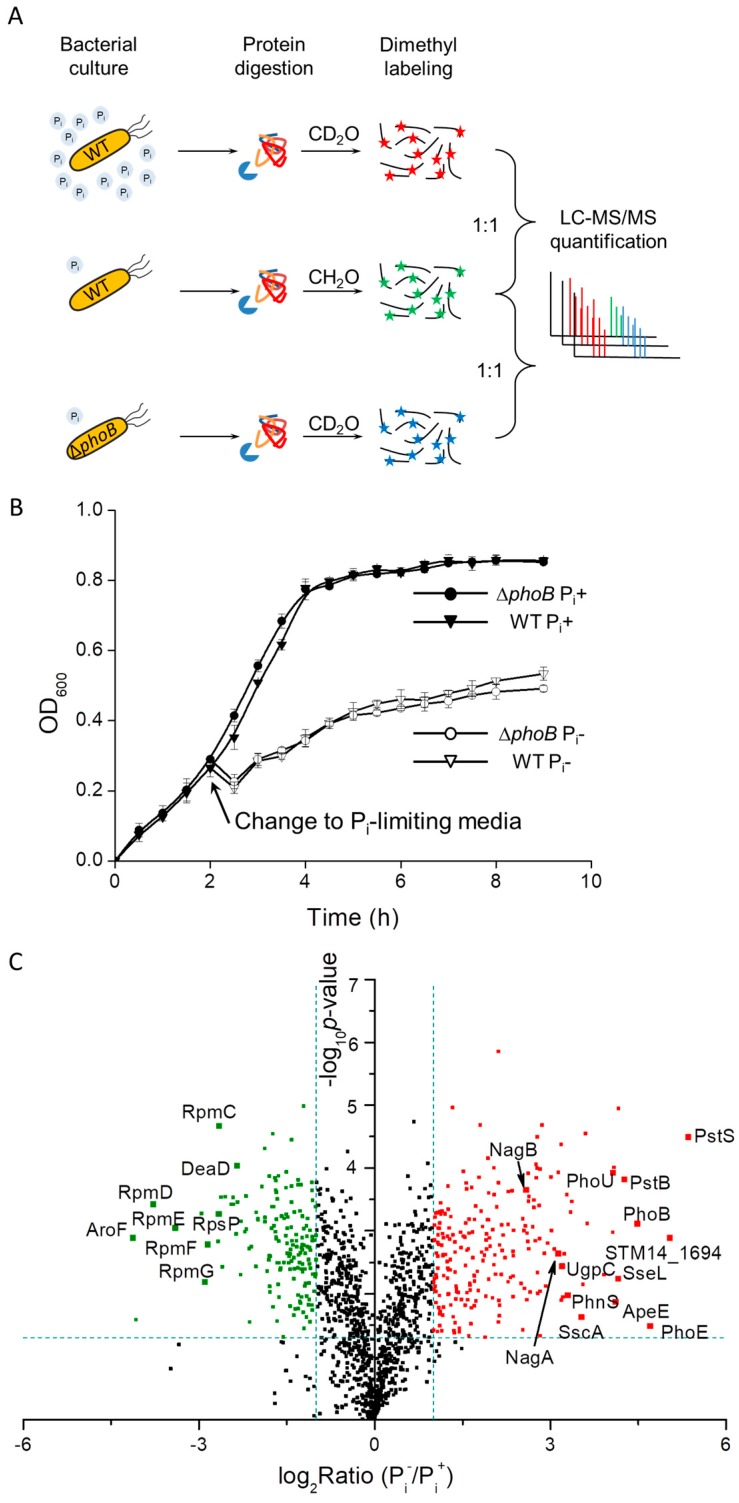
(**A**) A schematic diagram of experimental setup for quantitative profiling of *Salmonella* proteome under phosphate (P_i_)-limiting conditions. Protein samples from bacteria cultured in both P_i_-rich and P_i_-low media were digested, isotopically labeled, and equally mixed prior to LC-MS/MS analyses. (**B**) Bacterial growth curves of *Salmonella* WT (denoted by inverted triangles) and Δ*phoB* strains (denoted by circles) in MOPS media with or without the addition of 1 mM P_i_. For growth in P_i_-deficient conditions, bacteria were first cultured in P_i_-rich media until OD_600_ reached 0.3 and then the media was changed to MOPS without P_i_. The solid and open symbols represent P_i_^+^ and P_i_^−^ conditions respectively. (**C**) A volcano plot of detected *Salmonella* proteins under P_i_-rich (P_i_^+^) and P_i_^−^ low (P_i_^−^) conditions by LC-MS/MS. The logarithmic ratios of average fold changes are plotted on the x-axis. The y-axis plots negative logarithmic *p*-values from the *t*-test performed on three biological replicates. Dotted lines denote 2-fold (vertical) and *p* < 0.05 cutoff (horizontal). The up- or down-regulated proteins are denoted by the red and green dots, respectively.

To understand *S.* Typhimurium physiological responses to phosphate starvation, we quantitatively analyzed the bacterial proteome under P_i_-rich and P_i_-deficient conditions. Equivalent amounts of bacteria were harvested from different culturing conditions (see the method section for details) and used for quantitative LC-MS experiments. To further delineate PhoB-regulated proteins as well as PhoB-independent pathways upon the shift to P_i_ limitation, we also compared protein expression profile of a *phoB*-lacking mutant to that of the WT strain under P_i_-limiting conditions (Figure 1A). When the P_i_ supply is sufficient, the *phoB* mutant grew as well as the WT bacteria. Upon a shift to phosphate starvation, both bacterial strains exhibited much reduced growth rates and the growth of the *phoB* knockout mutant was not distinguishably slower than that of the WT (Figure 1B), suggesting the contribution of PhoB-independent pathways to bacterial replication. In other words, the lack of the Pho regulon did not noticeably inhibit bacterial growth under P_i_-poor conditions, which is consistent with previous findings [12].

For WT bacteria cultured under P_i_-replete and P_i_-deficient conditions, in total we identified 1651 *Salmonella* proteins (FDR < 1%) from three biological replicates. Upon the switch to phosphate starvation, indeed the bacterial proteome differed considerably as evidenced by 389 proteins of altered expression. Next, we graphed the fold ratios of protein abundance (P_i_^−^/P_i_^+^) as well as corresponding *p*-values in a volcano plot. Among those altered proteins, 225 proteins were up-regulated (shown in red) and 164 proteins were down-regulated (shown in green) under P_i_-limiting conditions (Figure 1C). For those highly induced proteins upon the shift to P_i_ starvation, we clearly observed several components of the PhoB regulon including PstS, PstB, PhoU, UgpC, PhnS and PhoE. The robust induction of classical PhoB-regulated proteins demonstrates the functional relevance of our culturing conditions. In contrast, the most depressed proteins (shown in the left in Figure 1C) under P_i_ deficiency are those associated with either the ribosome machinery or amino acid metabolism. A full list of the altered proteins is provided as Supplemental Table S1.

### 3.2. Protein-Protein Interaction Networks of Altered S. Typhimurium Proteins

**Figure 2:**
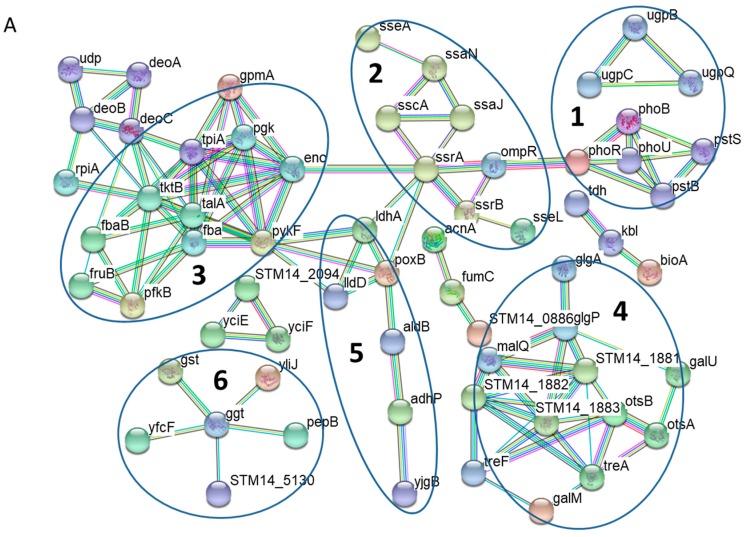

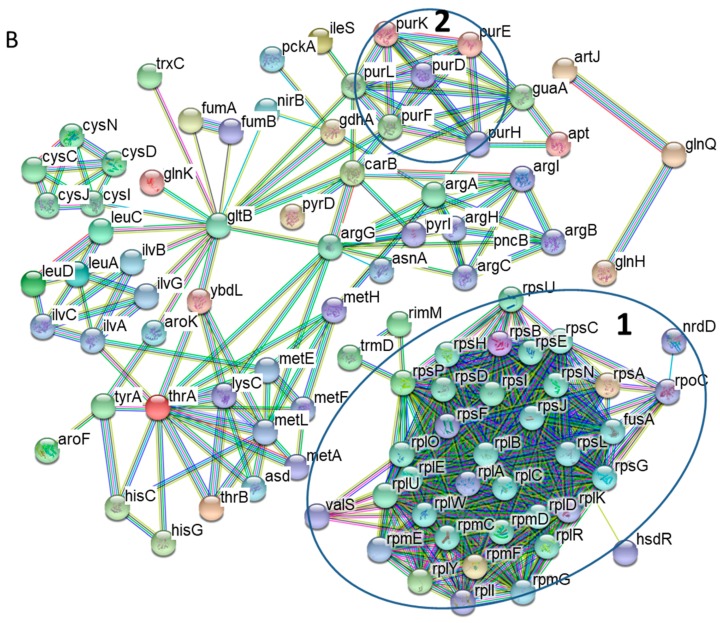
Network analyses of functional associations and/or interactions of those up-regulated proteins (**A**) and down-regulated proteins (**B**) during P_i_ starvation. Different clusters of interacting proteins were identified by using the STRING software with a highest confidence score. Some notable protein clusters are circled for the sake of clarity. The color lines linking different proteins represent the types of evidence (red line: fusion evidence; green line: neighborhood evidence; blue line: co-occurrence evidence; purple line: experimental evidence; yellow line: text-mining evidence; light blue line: database evidence; black line; co-expression evidence).

To examine protein-protein interactions among those differentially expressed proteins upon the shift to P_i_ starvation, we conducted network analysis by using the STRING database (Figure 2A). Several network features were revealed among the induced proteins during phosphate starvation. As expected, at least two clusters (enclosed in Circle 1) are readily visible corresponding to the classical Pho regulon. One network contains the PhoR-PhoB regulatory circuit and the Pst high affinity P_i_ transport system, whereas the small one comprises the Ugp uptake system (UgpB, UgpC and UgpQ) for glycerol-3-phosphate, a phosphorous-containing compound. Additionally, a prominent network is formed by several proteins associated with bacterial virulence (in Circle 2). *S.* Typhimurium possesses two distinct type III secretion systems (T3SSs) within its pathogenicity islands 1 and 2 (SPI-1 and SPI-2), which are essential for bacterial virulence [6]. Our data revealed a cluster of virulence proteins exclusively from SPI-2 T3SS, including SseA, SsaN, SscA, SsaJ, SsrA, SsrB, and SseL.

Many clusters formed by the up-regulated proteins are involved in bacterial energy metabolism. For instance, many proteins participating in glycolysis were induced under P_i_ starvation together with the pentose phosphate pathway, making a sizeable interaction network (in Circle 3). Furthermore, this bulky cluster encompasses the proteins involved in pyrimidine degradation such as DeoA, DeoB and DeoC, which provides glyceraldehyde 3-phosphate, a compound that can be readily fed into glycolysis. Interestingly, another notable network is defined by the proteins associated with glycogen and trehalose metabolism (in Circle 4). As the induction of trehalose metabolism during P_i_ limitation has not been noted before and the *otsB* gene encodes trehalose-phosphate phosphatase, next we sought to determine its contribution to *S.* Typhimurium adaptation to P_i_ deficiency. We constructed a mutant strain lacking *otsB* (Δ*otsB*) and measured the competition index when Δ*otsB* was co-cultured with its parental strain under P_i_-limiting conditions. The mutant showed no growth defect compared to its isogenic parental strain (see Supplementary Figure S2), suggesting that trehalose metabolism is unlikely to play a major role in coping with P_i_ starvation. Other clusters include several dehydrogenases (LdhA, LldD, PoxB, AldB, AdhP and YjgB in Circle 5) and proteins involved in glutathione transport and oxidation (Circle 6). 

With respect to those down-regulated proteins, a dominant interaction network is made of protein components of the ribosome machinery (Figure 2B, in Circle 1), indicating strong repression of protein translation. In addition, several clusters (scattered throughout the graph) are defined by the proteins involved in amino acid metabolism. Our data revealed down-regulation of the transport and biosynthetic pathways for several amino acids including leucine, isoleucine, methionine, histidine, serine, and arginine. Moreover, an interaction network is formed by the proteins in the purine biosynthesis pathway (in Circle 2).

### 3.3. Assignment of PhoB-regulated and PhoB-independent Pathways under P_i_ Starvation

**Figure 3:**
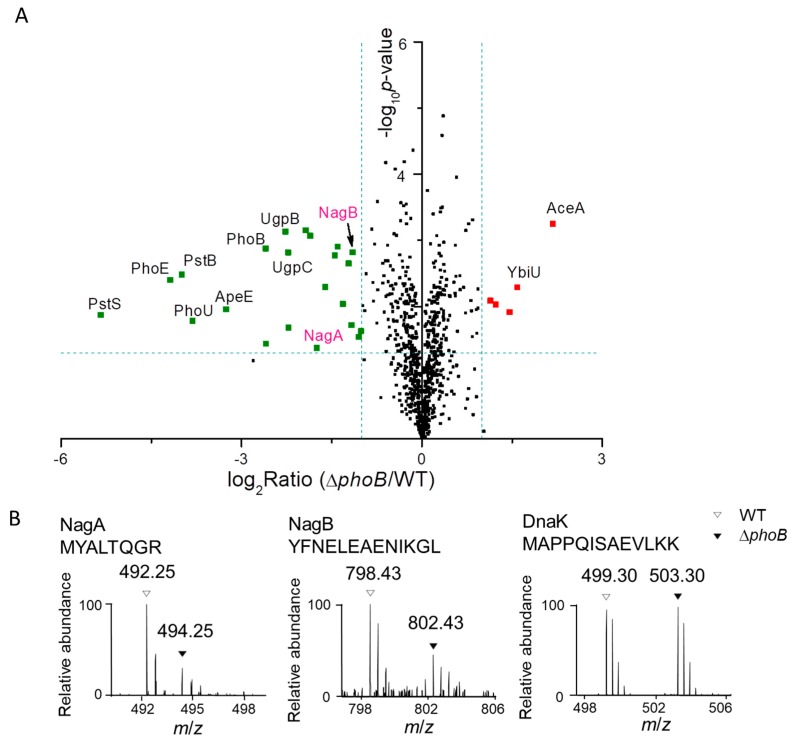

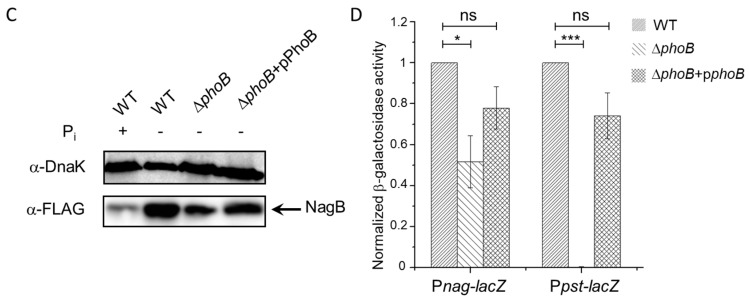
(**A**) A volcano plot of detected proteins in *Salmonella* WT and the *phoB* mutant strains by LC-MS/MS. The logarithmic ratios of average fold changes are plotted on the x-axis. The y-axis plots negative logarithmic *p*-values from the *t*-test performed on three biological replicates. Dotted lines denote 2-fold (vertical) and *p* < 0.05 cutoff (horizontal). The up- or down-regulated proteins are denoted by the red and green dots, respectively. (**B**) Representative mass spectra of dimethyl labeled peptides from NagA, NagB and DnaK (as a loading control). Peptides in WT and Δ*phoB* samples with light and heavy labels are indicated by open and filled triangles respectively. (**C**) Immunoblotting analyses of 3×FLAG-tagged NagB in *Salmonella* WT, Δ*phoB* and the complementation Δ*phoB +* pPhoB strains. DnaK was used as a loading control. (**D**) β-galactosidase activities of various reporter strains harboring the *lacZ* fusions to either the *nag* or the *pst* promoter (P*_nag_-lacZ* and P*_pst_-lacZ*, respectively) in WT, Δ*phoB* and Δ*phoB* + pPhoB backgrounds. Asterisks represent the range of *p*-values calculated by Student’s *t* test (*, *p* < 0.05; ***, *p* < 0.001; ns, not significant).

Given the central role of the PhoR-PhoB TCS in mediating *Salmonella* responses to P_i_ limitation, next we examined protein expression of a *phoB* knockout mutant in comparison to that of the WT strain under low P_i_ conditions. We found strikingly similar expression profiles of the two proteomes (Figure 3A). In fact, only a small set of proteins differed between the mutant and the WT strain, including 5 up-regulated (shown in red) and 22 down-regulated (shown in green) proteins (Figure 3A). These results contrast with the previous observation that the WT proteome differed vastly upon the switch from P_i_-replete to P_i_-limiting conditions (Figure 1C). Taken together, these findings suggest that the PhoR-PhoB regulatory system only mediates a rather small fraction of the altered proteome upon P_i_ starvation. In other words, many differentially expressed proteins are likely independent of the PhoR-PhoB control circuit. 

Next, we focused our attention on the subset of those down-regulated proteins in the *phoB* mutant relative to its parental strain, given that most proteins of the Pho regulon are PhoB-activated. As shown in Figure 3A, many known components of the Pho regulon were severely suppressed including proteins encoded in the *pst* and the *ugp* operons, PhnS (a 2-aminoethylphosphonate transporter), PhoE (an outer membrane phosphoporin), and ApeE (an outer membrane esterase), which is in good agreement with the well-documented activation of these proteins by PhoB [3]. Intriguingly, we also found marked repression of NagA and NagB, two key enzymes required for bacterial *N*-acetylglucosamine catabolism, in the *phoB* mutant proteome (Figure 3A). As also shown by the intensities of two representative peptides, the levels of NagA and NagB were 2 to 3-fold lower in the *phoB* mutant relative to its parental strain (Figure 3B). The *nag* operon encodes most proteins in the *N*-acetylglucosamine catabolic pathway. In *E. coli*, *N*-acetylglucosamine can be used as a carbon source after deacetylation by NagA and deamination by NagB, generating fructose 6-phosphate, an important glycolytic intermediate [27]. Encoded in the same operon, NagD is annotated as a phosphatase (potentially for fructose 6-phosphate) [28,29,30], though it was not detected in our work. Therefore, *N*-acetylglucosamine catabolism may serve as an additional means for bacterial P_i_ supply. In support of this notion, we found robust induction (5 to 9-fold) of NagA and NagB in WT bacteria upon the shift from P_i_-rich to P_i_-low conditions. 

### 3.4. PhoB-Dependent Induction of S. Typhimurium Nag during P_i_ Starvation

Considering the potential role of *N*-acetylglucosamine catabolism in generating additional P_i_, next we set out to verify the proteomic changes of NagB during P_i_ starvation by immunoblotting assays. To do this, we engineered *S.* Typhimurium strains chromosomally expressing a 3×FLAG-tagged version of NagB in different genetic backgrounds (WT, Δ*phoB* and a complementation strain Δ*phoB* + pPhoB). We confirmed the expression of PhoB in the WT and complementation strains but not the deletion mutant by using selected reaction monitoring (SRM) experiments (data provided as Supplementary Figure S3). Consistent with our proteomic data, immunoblotting analyses confirmed the marked elevation of NagB expression upon the switch to P_i_-limiting conditions. Importantly, the full induction of NagB required the regulator PhoB as evidenced by the lower level of NagB in a *phoB* knockout mutant. Furthermore, introduction of a copy of plasmid-borne *phoB* into the *phoB*-deleting strain restored, at least partially, the expression level of NagB during P_i_ deficiency. Together, these data suggest that *S.* Typhimurium up-regulates *N*-acetylglucosamine catabolism during P_i_ starvation in a PhoB-dependent manner. That being said, our immunoblotting data revealed partial induction of NagB in the *phoB*-lacking strain upon the shift to P_i_ limitation (Figure 3C), indicating potential PhoB-independent regulatory mechanisms as well. 

Next, we used β-galactosidase assays to clarify the transcriptional control of the *nag* operon by PhoB. We constructed *S.* Typhimurium reporter strains harboring *lacZ* fusions to the *nagB* promoter (P*_nag_*-*lacZ*) in different genetic backgrounds (WT, Δ*phoB* and Δ*phoB* + pPhoB). At the same time, we included bacterial strains harboring a plasmid with the *pst* promoter-*lacZ* fusion (P*_pst_*-*lacZ*) as positive controls in the assays. Measurements of β-galactosidase activities revealed that the Δ*phoB* strain exhibited markedly lower transcription levels of *lacZ* than those in the WT during P_i_ starvation (Figure 3D). Complementation with a *phoB*-expressing plasmid restored, at least in part, the *nag* promoter activity. These transcriptional readouts are indeed consistent with our previous proteomic and immunoblotting results. In comparison, the dependence of P*_pst_-lacZ* transcription on PhoB is much more pronounced because β-galactosidase activities were barely detected in the *phoB* deletion mutant. Taken together, these findings established that *S.* Typhimurium PhoB transcriptionally controls, at least partially, the induction of *N*-acetylglucosamine catabolism after the shift to P_i_ starvation.

## 4. Discussion

Previously, studies of bacterial adaptations to P_i_ starvation were mostly carried out in *E. coli* [2,11,12,13,14]. Our current work comparatively analyzed *S.* Typhimurium proteome upon the switch from P_i_-rich to P_i_-low conditions. The induction of the classical Pho regulon and the repression of ribosomal proteins and amino acid metabolism are in line with previous transcriptome analysis in *E. coli* [11,12]. It is interesting to note that under P_i_ starvation *S.* Typhimurium may have entered the stationary phase earlier than usual (see Figure 1B). Therefore, some of the proteomic changes (e.g., the general repression of protein translation) may be attributed to the stationary phase response [11]. Furthermore, many proteins associated with SPI-2 T3SS were up-regulated, which is consistent with the activation of SPI-2 genes under low P_i_ conditions [31]. It is well known that SCVs (the compartment where *S.* Typhimurium resides intracellularly) are poor in P_i_, which serves as one of the environmental cues for SPI-2 activation. It is also interesting to note that Chekabab et al. reported activation of T3SS virulence genes in enterohemorrhagic *E. coli* (EHEC) in response to P_i_ starvation [12]. 

In our study, many up-regulated *S.* Typhimurium proteins after the shift to P_i_-limiting conditions are involved in energy metabolism. Both glycolytic and pentose phosphate pathways (PPP) in central carbon metabolism were markedly induced, consistent with *E. coli* mRNA studies [11,12,14]. Previously, Schuhmacher et al. proposed that in *E. coli* ATP generation (from ATP synthase) would be adversely affected under P_i_ starvation, leading to increased substrate demand and hence higher glucose uptake [14]. Such a notion seems to explain well the up-regulation of glycolysis upon P_i_ limitation. It is also worthwhile to note that elevated glycolysis itself can serve as an additional means of ATP generation (though with less efficiency). In addition to glycolysis and PPP, pyrimidine degradation, glycogen and trehalose metabolism were induced as well. 

**Figure 4:**
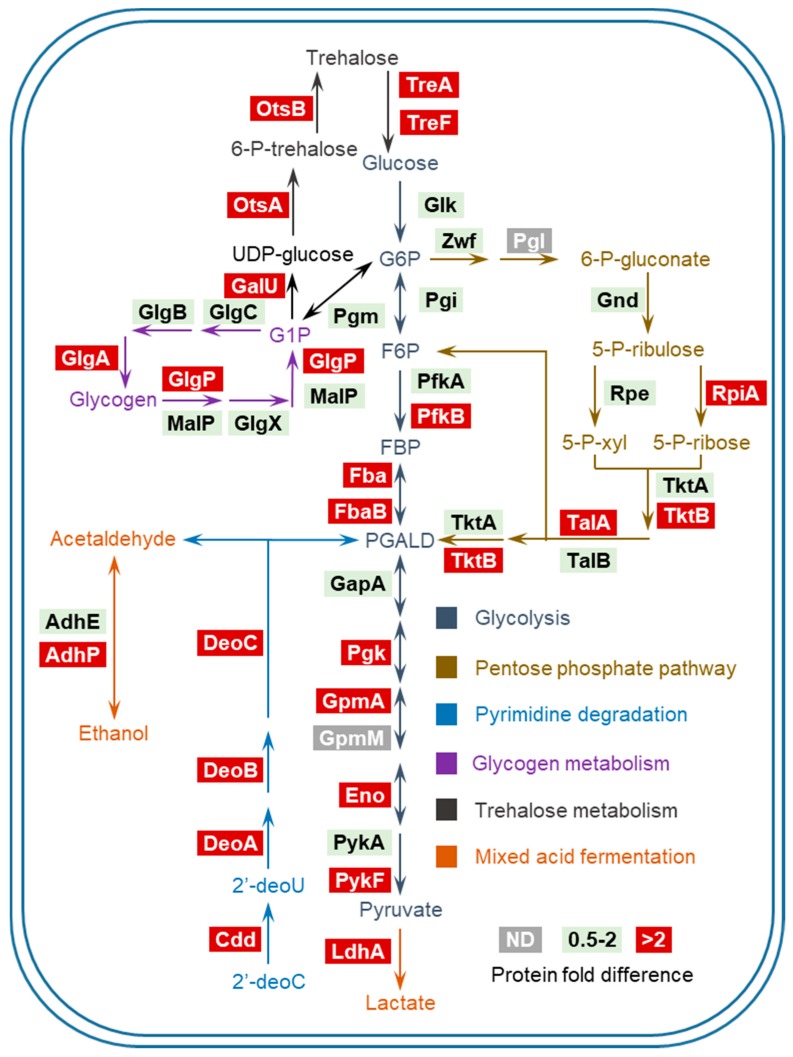
A pathway overview of *Salmonella* carbon metabolism under P_i_ starvation. ND: not detected. Bacterial enzymes are grouped by individual pathways with color-coded metabolites and arrows denoting the directionality of enzymatic reactions. Protein fold difference of those enzymes upon P_i_ starvation is color-coded as well.

To get a better understanding of these altered metabolic processes, we organized them in distinct modules containing sequential enzymatic reactions, generating a graphic overview of these pathways (Figure 4). Notably, the products of pyrimidine/glycogen/trehalose degradation pathways can be channeled into glycolysis at certain steps, indicating the extensive coupling of these up-regulated processes. It is worth noting that several metabolic pathways in Figure 4 (i.e., glycolysis, PPP and pyrimidine degradation) were found to be induced or at high expression levels during bacterial infection of host cells [9], suggesting that some proteomic features of intracellular *Salmonella* are likely to be shaped by P_i_ starvation inside SCVs. Other than substrates such as pyruvate, increased glycolysis also results in a higher level of NADH. In addition, NADH buildup would be intensified by the disruption of oxidative phosphorylation (due to the lack of P_i_). To counteract the excess of NADH, cells may resort to less efficient dehydrogenases rather than the electron transport chain [14]. In support of this notion, we found up-regulation of several dehydrogenases under P_i_-deficient conditions. 

Importantly, we also uncovered NagA and NagB as the additional members of the Pho regulon by comparing the proteome of a *phoB* mutant to that of the WT under P_i_-limiting conditions. We provided multiple lines of evidence including immunoblotting and β-galactosidase assays that Nag expression is under the control of PhoB during P_i_ starvation. Yet we do note that unlike classical PhoB-regulated proteins (e.g., those encoded in the *pst* operon), additional mechanisms seem to be involved in the control of Nag expression as well. A known repressor of the *nag* operon is NagC, which unfortunately was not detected in our LC-MS experiments probably owing to its low expression level. Last but not the least, the proteomic study of the *phoB* mutant allows us to conclude that PhoB only regulates a small subset of the altered proteins upon the shift from P_i_-rich to P_i_-poor conditions. In other words, most proteomic changes we observed under P_i_ starvation are PhoB-independent, including those carbon metabolic pathways discussed above. 

## Supplementary Materials

The following are available online, Figure S1: A representative gel image of bacterial cell lysates fractionated by SDS-PAGE, Figure S2: Competitive growth index of the WT and Δ*otsB* strains, Figure S3: Determination of PhoB levels in WT, Δ*phoB* and Δ*phoB* + pPhoB strains by selected reaction monitoring (SRM) experiments, Table S1: A full list of protein identifications as well as the altered proteins.
