# Supplementary material for: A Proteomic View of Salmonella Typhimurium in Response to Phosphate Limitation
Source: Proteomes. 2018 Apr 25;6(2):19. doi: 10.3390/proteomes6020019 (PMC6027262; doi:10.3390/proteomes6020019)

**Figure S1.** A representative gel image of bacterial cell lysates fractionated by SDS-PAGE. The entire gel was equally divided into 8 fractions (denoted by the dotted lines) for subsequent in-gel protein digestion.


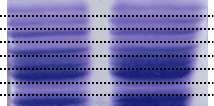


**Figure S2.** Competitive growth index of the WT and Δ*otsB* strains that were co-cultured under P_i_-limiting conditions. The competitive index is defined by the ratio of the number of WT colonies to that of Δ*otsB* colonies.


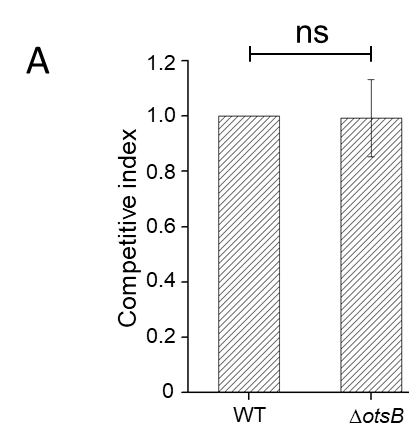


**Figure S3.** Determination of PhoB levels in WT, Δ*phoB* and Δ*phoB*+pPhoB strains by selected reaction monitoring (SRM) experiments. Bacterial samples from the WT, Δ*phoB* and Δ*phoB*+pPhoB strains were gel-fractionated, digested and analyzed by LC-MS. To quantify PhoB, a transition of 557.8→443.6 (DIPVVMLTAR) was used. For intensity normalization, a transition of 401.24→601.37 (TVGAGVVAK) from the elongation factor Tuf_2 was used. Corresponding peak areas for these transitions were obtained from Thermo Xcalibur 2.2.


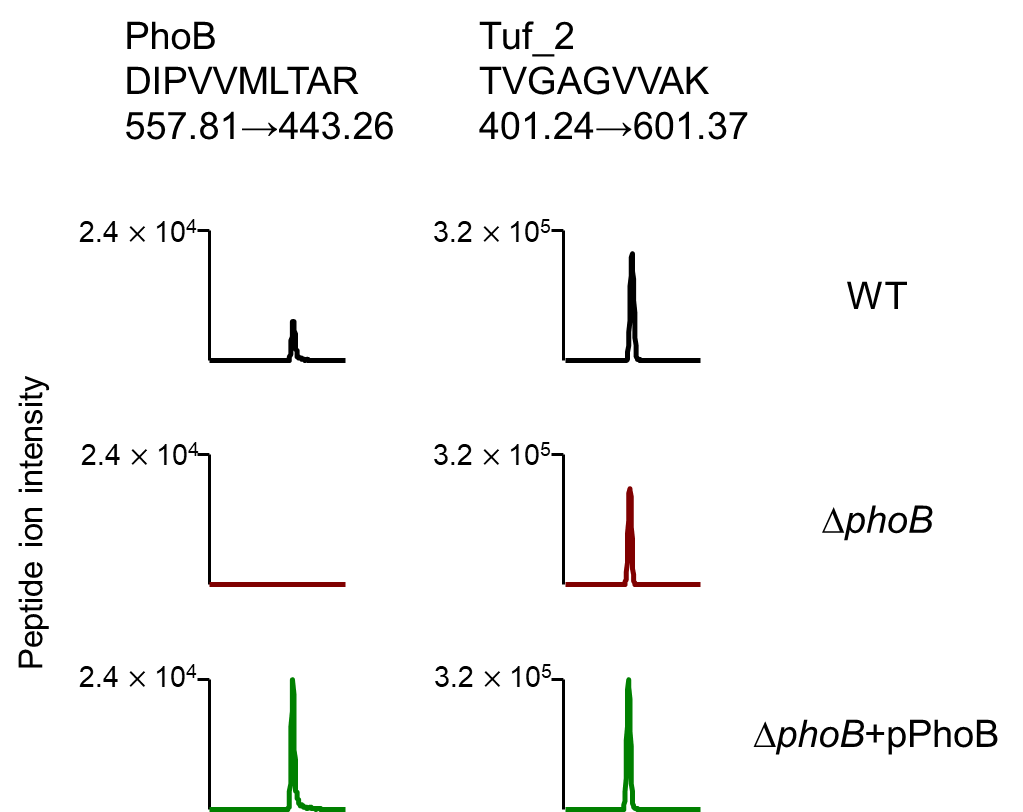

Supplement: Supplementary file 1 [file proteomes-06-00019-s001.zip › Supplementary+Figures.docx]
